# Interspecies relationships between nosocomial pathogens associated to preterm infants and lactic acid bacteria in dual-species biofilms

**DOI:** 10.3389/fcimb.2022.1038253

**Published:** 2022-10-17

**Authors:** Josué Jara, Rubén Jurado, Víctor G. Almendro-Vedia, Iván López-Montero, Leonides Fernández, Juan Miguel Rodríguez, Belén Orgaz

**Affiliations:** ^1^ Departamento de Nutrición y Ciencia de los Alimentos, Facultad de Veterinaria, Universidad Complutense de Madrid, Madrid, Spain; ^2^ Departamento de Farmacia Galénica y Tecnología de los Alimentos, Facultad de Veterinaria, Universidad Complutense de Madrid, Madrid, Spain; ^3^ Departamento de Química Física, Facultad de Químicas, Universidad Complutense de Madrid, Madrid, Spain; ^4^ Instituto de Investigación Biomédica Hospital Doce de Octubre (imas12), Madrid, Spain; ^5^ Instituto Pluridisciplinar, Universidad Complutense de Madrid, Madrid, Spain

**Keywords:** biofilms, nasogastric enteral feeding tubes, lactic acid bacteria, *klebsiella pneumoniae*, *Serratia marcescens*, *Staphylococcus aureus*, *Staphylococcus epidermidis*, preterm infants

## Abstract

The nasogastric enteral feeding tubes (NEFTs) used to feed preterm infants are commonly colonized by bacteria with the ability to form complex biofilms in their inner surfaces. Among them, staphylococci (mainly *Staphylococcus epidermidis* and *Staphylococcus aureus*) and some species belonging to the Family *Enterobacteriaceae* are of special concern since they can cause nosocomial infections in this population. NETF-associated biofilms can also include lactic acid bacteria (LAB), with the ability to compete with pathogenic species for nutrients and space. Ecological interactions among the main colonizers of these devices have not been explored yet; however, such approach could guide future strategies involving the pre-coating of the inner surfaces of NEFTs with well adapted LAB strains in order to reduce the rates of nosocomial infections in neonatal intensive care units (NICUs). In this context, this work implied the formation of dual-species biofilms involving one LAB strain (either *Ligilactobacillus salivarius* 20SNG2 or *Limosilactobacillus reuteri* 7SNG3) and one nosocomial strain (either *Klebsiella pneumoniae* 9SNG3, *Serratia marcescens* 10SNG3, *Staphylococcus aureus* 45SNG3 or *Staphylococcus epidermidis* 46SNG3). The six strains used in this study had been isolated from the inner surface of NEFTs. Changes in adhesion ability of the pathogens were characterized using a culturomic approach. Species interactions and structural changes of the resulting biofilms were analyzed using scanning electron microscopy (SEM) and confocal laser scanning microscopy (CLSM). No aggregation was observed in dual-species biofilms between any of the two LAB strains and either *K. pneumoniae* 9SNG3 or *S. marcescens* 10SNG3. In addition, biofilm thickness and volume were reduced, suggesting that both LAB strains can control the capacity to form biofilms of these enterobacteria. In contrast, a positive ecological relationship was observed in the combination *L. reuteri* 7SNG3-*S. aureus* 45SNG3. This relationship was accompanied by a stimulation of *S. aureus* matrix production when compared with its respective monospecies biofilm. The knowledge provided by this study may guide the selection of potentially probiotic strains that share the same niche with nosocomial pathogens, enabling the establishment of a healthier microbial community inside NEFTs.

## Introduction

Bacterial biofilms are surface-associated communities embedded in a matrix of self-producing extracellular polymeric substances (EPS) (Costerton, 1995; Vert et al., 2012; Flemming et al., 2016b). Polymicrobial biofilms are behind most of the persistent infections related to the use of clinical devices (Høiby et al., 2011; Lamas et al., 2018). Their inner surfaces can act as a reservoir of nosocomial microorganisms to the host. Microorganisms are sheltered inside these devices and benefited from multiple advantages; the close cell-to-cell contact facilitates gene exchange while the presence of the matrix interferes with the host immunity mechanisms, increases tolerance to antimicrobials and provides mechanical stability to the community (Flemming et al., 2016a; Koo and Yamada, 2016; Hou et al., 2018; Karygianni et al., 2020; Flemming et al., 2021; Jara et al., 2021).

The rapid formation of biofilms in the inner surface of the nasogastric enteral feeding tubes (NEFTs) used for the feeding of preterm infants seems particularly worrying (Bussy et al., 1992; Mehall et al., 2002; Liu et al., 2014; Gómez et al., 2016a; Ogrodzki et al., 2017). Prematurity is the main cause of neonatal deaths in children under the age of two (WHO, 2022), and nosocomial infections and sepsis are responsible for a high percentage of preterm-associated morbidity and mortality (ECDC, 2018). Although use of NEFTS is critical for the survival of many preterm infants, the formation of biofilms inside these devices represents a risk factor for the acquisition of nosocomial infections as they may allow the entry and replication of hospital-associated microorganisms (Mehall et al., 2002; Gómez et al., 2016b; Ogrodzki et al., 2017).

The nosocomial microbes most frequently isolated from NEFT-associated biofilms include Gram-negative (*Klebsiella*, *Serratia* and related enterobacteria) and Gram-positive (*Staphylococcus*, *Enterococcus*) bacteria. They can arise from a wide variety of sources including the own host, their relatives, the feeding (in the case of own mother`s milk) or the hospital staff and environment (Mathus-Vliegen et al., 2006; Moles et al., 2013; Cong et al., 2016; Gómez et al., 2016a; Petersen et al., 2016). *Staphylococcus epidermidis* and *Staphylococcus aureus* are the most abundant species isolated from NEFTs (Jara et al., 2021), and are also frequently implicated in catheter- and prosthetic devices-associated infections due to their ability to form biofilms (Cruz et al., 2018; Cassini et al., 2019; Davidson et al., 2019; Monegro et al., 2020; Kranjec et al., 2021). Previous studies have shown that their presence in a surface may promote the adhesion and proliferation of other bacterial species, including Gram-negative ones (Puga et al., 2018; Jara et al., 2020). Among the later, the presence of *Klebsiella pneumoniae* and *Serratia marcescens* is specially concerning in neonatal intensive care units (NICUs) since both species are opportunistic pathogens and have been related with respiratory and urinary tract infections and sepsis in preterm infants (Dessì et al., 2009; Mahlen, 2011; Singhai et al., 2012; Gonzalez et al., 2013; Vuotto et al., 2014; Shokouhfard et al., 2015; Satpathy et al., 2016; Srinivasan et al., 2016; Piperaki et al., 2017; Vuotto et al., 2017; Cristina et al., 2019). Prophylactic and metaphylactic use of antibiotics is a widespread practice in neonatal intensive care units (NICUs) in order to prevent sepsis and infections (ECDC, 2018). However, the burden of antimicrobial resistance is particularly high among NICUs isolates belonging to the species cited above and, therefore, there is a need for alternative strategies to prevent or minimize the presence of these species in NEFTs-associated biofilms.

Although less frequently, some lactic acid bacteria (LAB), including strains belonging to species with a “qualified presumption of safety” (QPS) status (EFSA, 2018), can also be isolated from NEFTs (Gómez et al., 2016a; Petersen et al., 2016; Jara et al., 2021), indicating an adaptation to live in these hospital-related devices. Therefore, such strains would be good candidates for the pre-coating of NEFTs in order to minimize the adhesion of nosocomial pathogens. Some studies have evaluated the efficacy of some LAB and bifidobacterial strains to compete with potentially pathogenic species that share the same niche (Velraeds et al., 2000; Gomaa, 2013; Vuotto et al., 2014; Bossa et al., 2017; Esaiassen et al., 2018; Jara et al., 2020). Understanding species interactions in complex microbial communities would be useful to develop probiotic-based strategies specifically designed for the requirements of hospitalized preterm infants. In this context, the main objective of this study was to evaluate the interspecies interactions in dual biofilms involving one LAB strain (either *L. salivarius* 20SNG2 or *L. reuteri* 7SNG3) and one nosocomial strain (either *K. pneumoniae* 9SNG3, *S. marcescens* 10SNG3, *S. aureus* 45SNG3 or *S. epidermidis* 46SNG3).

## Material and methods

### Bacterial strains and growth conditions

Two lactic acid bacteria (LAB), *L. salivarius* 20SNG2 and *L. reuteri* 7SNG3, and four nosocomial pathogens, (*K. pneumoniae* 9SNG3*, S. marcescens* 10SNG3*, S. epidermidis* 46SNG3 and *S. aureus* 45SNG3) were used in this study. All of them were isolated in a previous work from the inner surface of NEFTs that were inserted into preterm infants for approximately 48 h (Jara et al., 2021). De Man Rogosa and Sharpe (MRS, Oxoid, Basingstoke, UK) medium was routinely used for the growth of the LAB strains and Brain Heart Infusion (BHI, Oxoid; Basingstone, UK) for the rest of the strains. For cultivation, an isolated colony of each strain was transferred into 10 mL of the corresponding culture media and incubated at 37°C overnight. Then, cells were washed twice by centrifugation at 17,000 × *g* for 10 min at 4°C, and suspended into 10 mL of the same culture medium. The optical density at 600 nm (OD_600_) of the bacterial suspensions was adjusted to 0.1 (~10^7^ cfu/mL).

### Antimicrobial susceptibility testing

The inhibitory capacity of the LAB strains against the four pathogenic strains was examined by an agar diffusion method. First, an isolated colony of each LAB strain was inoculated into tubes containing 10 mL of MRS and incubated overnight at 37°C. Then, 5 µL drops of these exponential phase LAB suspensions were deposited on the surface of MRS agar plates and incubated 24 h at 37°C. Finally, 9 mL tubes of soft BHI agar [0.75% (w/v) agar] at 55°C were inoculated with 100 µL of a bacterial suspension (~10^5^ cfu/mL) of each pathogen and poured into the plates previously seeded with the LAB drops. Once this agar layer was cooled down, plates were incubated overnight at 37°C. Finally, the inhibition halos were measured. Experiments were carried out in triplicate.

### Experimental system for biofilm development

Biofilms were growing in a batch system called carousel described by Orgaz et al. (2011). In this system, sixteen 22 *x* 22 mm microscope glass coverslips (Thermo Scientific, Germany) were inserted vertically into the narrow radial slits of a Teflon platform (6.6 cm diameter). The platform and its lid were assembled by an axial stainless steel rod for handling and placed into a 600 mL glass beaker (**Figure S1**). The whole system was heat-sterilized as a unit. Sixty mL of the corresponding culture medium were inoculated with 1 mL of the previously obtained cellular suspensions (10^7^ cfu/mL) to achieve a calibrated starting concentration of ~10^5^ cfu/mL. Monospecies LAB biofilms were developed in MRS culture medium (in parallel, LAB were grown in BHI to verify they reached similar numbers in both media) and BHI was used for monospecies biofilms of the four above mentioned pathogens. For dual species biofilms, 1 mL of each microbial suspension was inoculated to achieve a 1:1 proportion of both strains. In this case, BHI was used as culture medium. The system was incubated in aerobic conditions at 37°C for 48 h. Under these conditions, the developed biofilms have a submerged area and an air-liquid interphase (**Figure S1**).

### Biofilm cell recovery and counting

During incubation, two diametrically opposed coverslips were aseptically extracted from the platform with sterile tweezers and washed into 0.85% (w/v) saline to remove weakly attached cells. Both sides of the coverslip surface were thoroughly scraped using a sterile swab that was finally introduced into 1.5 mL of sterile peptone water. No adherent cells were removed after 1 min by vortexing the tubes at high speed. The resulting cellular suspension was decimally diluted in sterile peptone water and 50 µL of each dilution were plated onto MRS agar (selective counts of the LAB strains), MacConkey agar (MCK, Oxoid; Basingstone, UK) (selective counts of either *K. pneumoniae* 9SNG3 *or S. marcescens* 10SNG3-20) and/or Baird Parker agar (BP, Pronadisa, Spain) (staphylococcal counts), and incubated at 37°C for 24 h for enumeration.

### Biomass determination

For the quantification of attached biomass (cells plus matrix), biofilms were stained by immersion of the coverslips into 4 mL of a crystal violet solution (1% w/v) (Sigma Aldrich, Spain) for 2 min. This process was repeated twice, and the excess of the colorant was removed with sterile MilliQ water before drying. For image acquisition, coverslips were scanned using a HP Scanjet 300 at a resolution of 600 dpi. These samples were afterward immersed into 4 mL of ethanol for removing the stained biomass. The whole biomass was scraped from the surfaces with a sterile cell scraper (APTACA, Italy) and homogenized in ethanol. For biomass quantification, OD_595nm_ was measured in a spectrophotometer DIGILAB U-2800 (Hitachi, United Kingdom).

### Visualization of biofilms by Scanning Electron Microscopy

Scanning electron microscopy (SEM) images of the developed biofilms were acquired at the facilities of the Microscopy Center of the Complutense University of Madrid (Spain). For this purpose, biofilms grown in coverslips were firstly washed in 50 mL of saline solution 0.85% (w/v) and then fixed by immersion in a solution containing 4% paraformaldehyde (Sigma Aldrich, Spain) and 3% glutaraldehyde (Sigma Aldrich, Spain) in 0.1 M phosphate buffer solution (PBS) (pH 7.2) for 12 h at 4°C. After this step, samples were washed with MilliQ water and progressively dehydrated by passage through a graded series of ethanol solutions from 40% to 100%. Finally, the samples were critical point dehydrated in a Leica CPD300 (Leica, Germany) using carbon dioxide as the transition fluid and coated with gold-palladium in an automated sputter coater Leica EM ACE200 (Leica, Germany). Samples were examined under a JEOL 6400 JSM electron microscope.

### Visualization of biofilms by confocal laser scanning microscopy

Developed biofilms were washed into a tube containing 50 mL of sterile saline solution 0.85% (w/v) and stained using SYTO13 (Invitrogen™, Massachusetts, USA). Series of xy images with a z-step of 1 µm when then acquired using a Nikon ECLIPSE Ti microscope (Software NIS Elements, 4.5.1.01 edition) equipped with a Nikon C2 confocal scanning module, 488 and 561 nm continuous lasers, emission bandpass filters and an oil immersion 60*X* objective. Three-dimensional reconstructions from z-stacks were carried out using IMARIS 8.0 software (Bitplane, Zürich, Switzerland). To calculate the biovolume (μm^3^), the MeasurementPro module of this software was used. For this, four images of two coupons of each type of biofilm were analyzed.

### Statistical analysis

At least four independent experiments were performed to obtain mean values. Data were analyzed with Statgraphics Centurion software (Statistical Graphics Corporation, Rockville, Md., USA). One-way analysis of variance (ANOVA) was carried out to determine whether samples were different or not at a 95% confidence level (p< 0.05).

## Results

### Antimicrobial activity and structure of LAB monostrain biofilms

**Figure 1:**
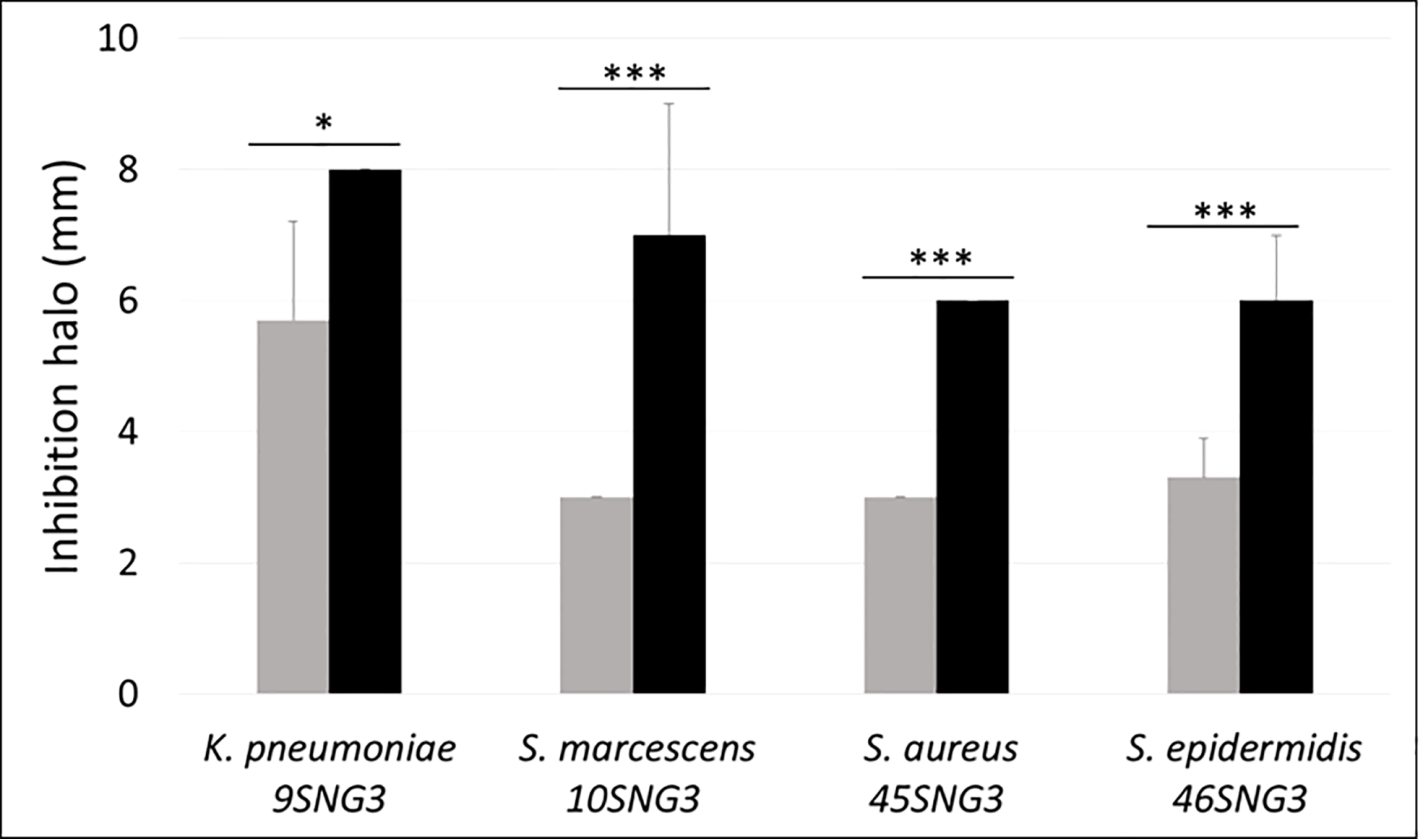
Antimicrobial activity (mm) of *L. salivarius* 20SNG2 (black bars) and *L. reuteri* 7SNG3 (grey bars) against the selected strains of *S. marcescens*, *K. pneumoniae*, *S. aureus* and *S. epidermidis*. Asterisks show statistical differences between the LAB effect on these strains: * (p < 0.05), and *** (p < 0.001).

Both LAB strains inhibited the growth of the four tested pathogens. The antimicrobial activity of *L. salivarius* 20SNG2 was significantly greater than that of *L. reuteri* 7SNG3 (**Figure 1**).

**Figure 2:**
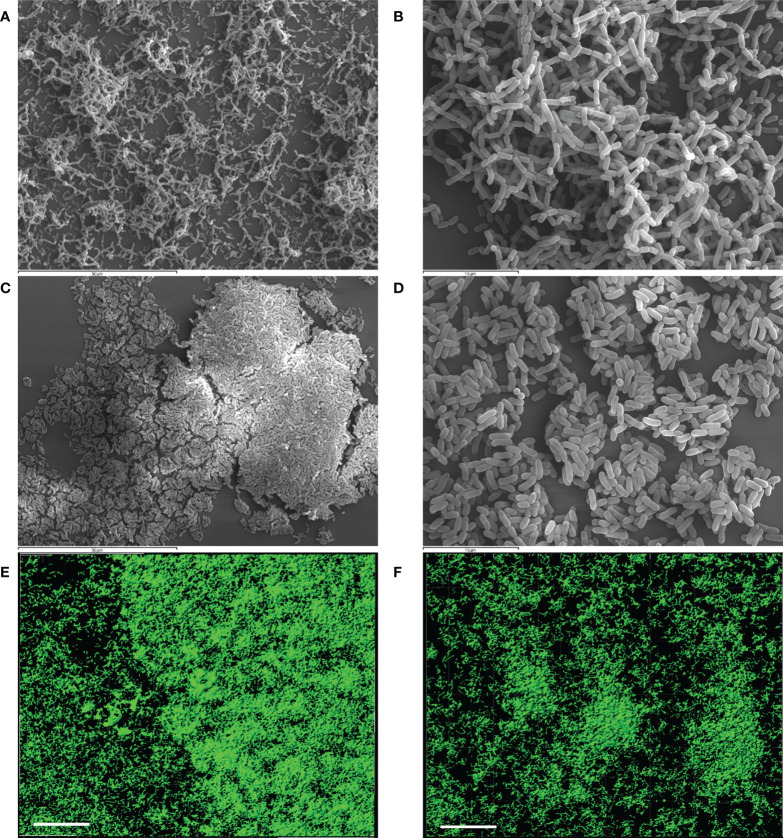
Representative images of biofilms (48 h) of *L. reuteri* 7SNG3 obtained by SEM at 1,000× **(A)** and 3,000× **(B)**, and by CLSM **(E)**. Representative images of biofilms (48 h) of *L. salivarius* 20SNG2 biofilms obtained by SEM at 1,000× **(C)** and 3,000× **(D)**, and by CLSM **(F)**. For CLSM image acquisition, cells (in green) were stained with SYTO 13. CLSM scale bar: 50 µm.

Structural features of the biofilms formed independently by each LAB strain after 48 h were analyzed by SEM and CLSM (**Figure 2**). SEM images of *L. reuteri* 7SNG3 biofilms showed densely packed cellular structures forming a multilayer network that covered most of the surface (**Figures 2A, B**), whereas *L. salivarius* 20SNG2 biofilms were characterized by the presence of big clusters scattered all over the surface (**Figures 2C, D**). High cellular density was accumulated in the liquid-air interphase observing in some cases mucous aggregates. Biovolume values for each strain were calculated from CLSM images, being on average 3.41 (± 1.21) × 10^5^ µm^3^ and 1.39 (± 0.27) × 10^5^ µm^3^ for *L. reuteri* 7SNG3 and *L. salivarius* 20SNG2, respectively (**Table 1**). The maximum width values of *L. reuteri* 7SNG3 and *L. salivarius* 20SNG2 biofilms were 12 µm and 9 µm, respectively.

**Table 1:** Biovolume (µm^3^) and maximum thickness (µm) of monospecies and dual-species biofilms of the microbial strains combinations used in this work.

Type of biofilm	Strains	Biovolume (µm^3^)	Maximun thickness (µm)
**Monospecies**	*L. reuteri* 7SNG3	3.41 ± [1.21] × 10^5^	12 ± 2
	*L. salivarius* 20SNG2	1.39 ± [0.27] × 10^5^	9 ± 1
	*K. pneumoniae* 9SNG3	4.56 ± [0.52] × 10^5^	20 ± 1
	*S. marcescens* 10SNG3	1.43 ± [0.07] × 10^5^	6 ± 0
	*S. aureus* 45SNG3	4.63 ± [2.47] × 10^4^	7 ± 3
	*S. epidermidis* 46SNG3	2.32 ± [2.09] × 10^4^	3 ± 1
**Dual-species**	*L. reuteri* 7SNG3 + *K. pneumoniae* 9SNG3	5.83 ± [2.27] × 10^5^	12 ± 2
	*L. reuteri* 7SNG3 + *S. marcescens* 10SNG3	1.33 ± [0.56] × 10^5^	6 ± 2
	*L. reuteri* 7SNG3 + *S. aureus* 45SNG3	1.11 ± [1.06] × 10^5^	10 ± 5
	*L. reuteri* 7SNG3 + *S. epidermidis* 46SNG3	1.36 ± [0.12] × 10^5^	10 ± 0
	*L. salivarius* 20SNG2 + *K. pneumoniae* 9SNG3	4.37 ± [5.91] × 10^5^	13 ± 3
	*L. salivarius* 20SNG2 + *S. marcescens* 10SNG3	1.24 ± [0.51] × 10^5^	8 ± 3
	*L. salivarius* 20SNG2 + *S. aureus* 45SNG3	2.94 ± [1.64] × 10^4^	4 ± 1
	*L. salivarius* 20SNG2 + *S. epidermidis* 46SNG3	5.66 ± [5.93] × 10^4^	6 ± 3

Both parameters are means ± SD of data from 8 images obtained after reconstructing z-stacks (4 image stacks from 2 independent biofilm samples).

### Formation of dual LAB-pathogen biofilms

**Figure 3:**
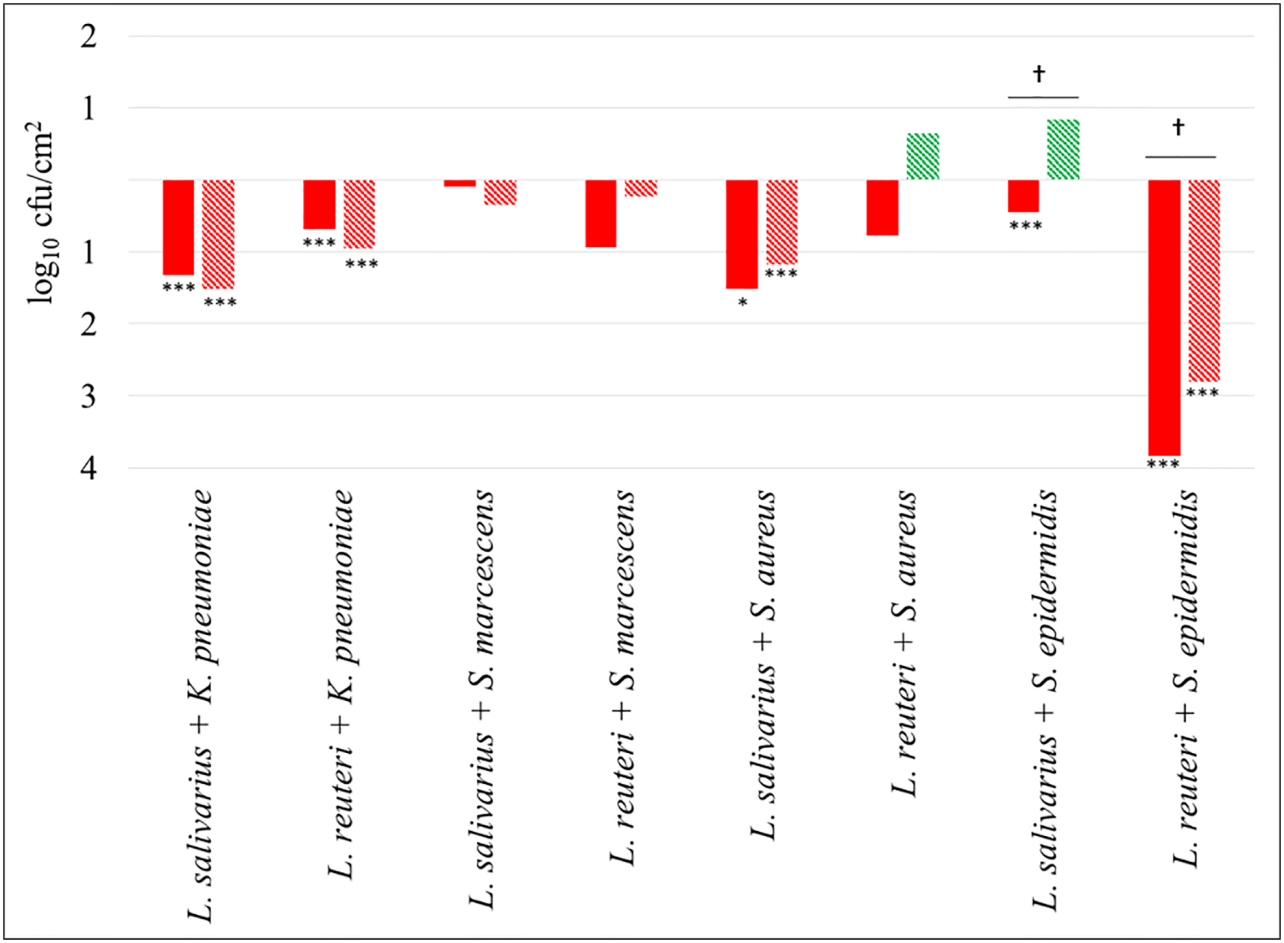
Changes in viable attached cell population (log_10_ cfu/cm^2^) in dual-species biofilms. Red and green bars mean log reduction or increase compared with monospecies biofilms of the respective pathogenic strains. Filled and lined bars represent 24 h and 48 h samples, respectively. Asterisks show statistical differences between monospecies pathogenic biofilms and dual-species biofilms with one of the LAB strains: * (p < 0.05) and *** (p < 0.001). † indicates statistical differences between 24 h and 48 h samples (n=4).

**Figure 3** shows differential biofilm formation when *K. pneumoniae* 9SNG3, *S. marcescens* 10SNG3, *S. aureus* 45SNG3 and *S. epidermidis* 46SNG3 were co-cultivated with either *L. salivarius* 20SNG3 or *L. reuteri* 7SNG3. In general, the ecological relationships in dual species biofilms were neutral or negative for the four pathogenic strains, although the impact was different depending on the LAB strain. In mixed biofilms with *L. salivarius* 20SNG2, *S. marcescens* 10SNG3 and *S. epidermidis* 46SNG3 were slightly (but not significantly) inhibited whereas there were statistically significant decreases (p<0.001) in the attached viable population of *K. pneumoniae* 9SNG3 and *S. aureus* 45SNG3 (log reductions of 1.32 and 1.52, respectively, at 24 h and 1.52 and 1.20, respectively, at 48 h) (**Figure 3**). In the case of *L. reuteri* 7SNG3, the population of attached *K. pneumoniae *9SNG3 was also significantly decreased (p<0.001) and, additionally, there was a strong reduction of the attached population of *S. epidermidis* 46SNG3 (log reductions of 3.83 after 24 h and 2.8 after 48 h, when compared with its corresponding monospecies biofilm). In contrast, the ability of biofilm formation of *S. marcescens* 10SNG3 and *S. aureus* 45SNG3 was not affected by the presence of *L. reuteri* 7SNG3 (**Figure 3**).

**Figure 4:**
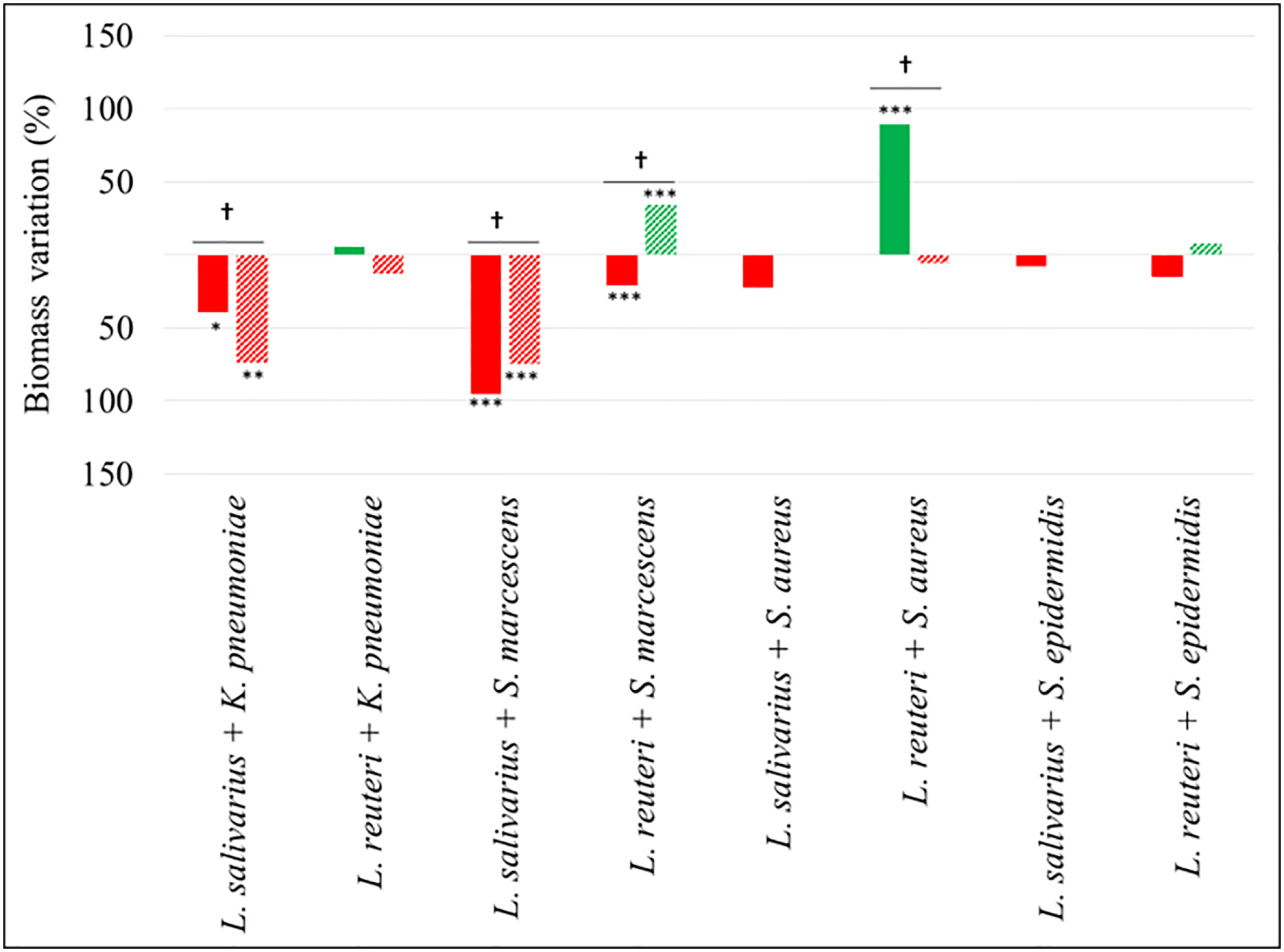
Percentage of biomass variation in dual-species biofilms. Red and green bars mean biomass reduction and biomass increasing compared with monospecies biofilms of the respective pathogenic strains. Filled and lined bars represent 24 h and 48 h samples, respectively. Asterisks show statistical differences between monospecies pathogenic biofilms and dual-species biofilms with both LAB: * (p < 0.05), ** (p < 0.005) and *** (p < 0.001). † indicate statistical differences between 24 h and 48 h samples (n=4).

The biomass (cells + matrix) produced as a consequence of the assayed interactions was also measured (**Figure 4**). Mixed biofilms of both enterobacterial strains with *L. salivarius* 20SNG2 revealed a significant decrease in biomass values compared with their respective monospecies biofilms, reaching up to a 95% reduction in the case of *S. marcescens* 10SNG3 (p<0.001) and a 74% reduction in that of *K. pneumoniae *9SNG3 (p<0.01). In contrast, significantly higher biomass values (p<0.001) were observed in mixed biofilms of *L. reuteri* 7SNG3 with *S. aureus* 45SNG3.

### Structure of dual LAB-pathogen biofilms

**Figure 5:**
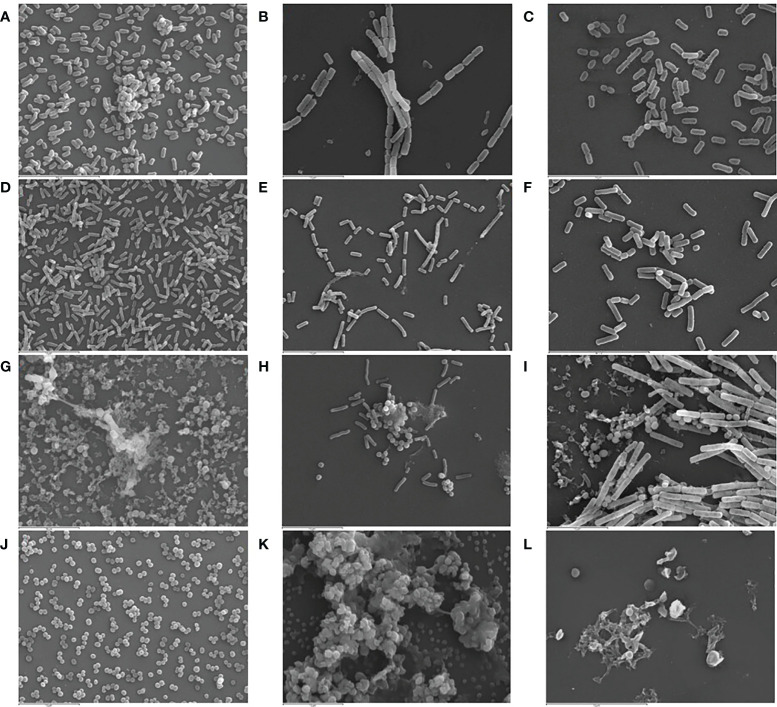
Representative SEM images (3,000×) of monospecies biofilms (48 h) of *K. pneumoniae* 9SNG3 **(A)**, *S. marcescens* 10SNG3 **(D)**, *S. aureus* 45SNG3 **(G)** and *S. epidermidis* 46SNG3 **(J)**. Representative SEM images of dual-species biofilms of *K. pneumoniae* 9SNG3 **(B)**, *S. marcescens* 10SNG3 **(E)**, *S. aureus* 45SNG3 **(H)** and *S. epidermidis* 46SNG3 **(K)** with *L. reuteri* 7SNG3. Representative SEM images of dual-species biofilms of *K. pneumoniae* 9SNG3 **(C)**, *S. marcescens* 10SNG3 **(F)**, *S. aureus* 45SNG3 **(I)** and *S. epidermidis* 46SNG3 **(L)** with *L. salivarius* 20SNG2.

**Figure 6:**
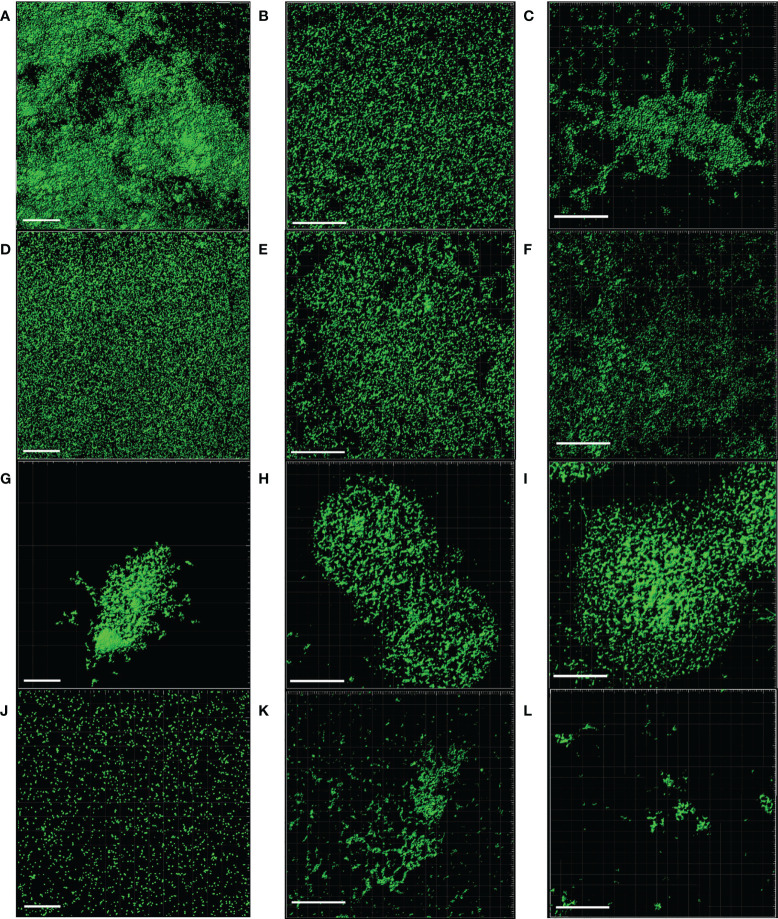
Representative CLSM images of monospecies biofilms (48 h) of *K. pneumoniae* 9SNG3 **(A)**, *S. marcescens* 10SNG3 **(D)**, *S. aureus* 45SNG3 **(G)** and *S. epidermidis* 46SNG3 **(J)**. Representative CLSM images of dual-species biofilms of *K. pneumoniae* 9SNG3 **(B)**, *S. marcescens* 10SNG3 **(E)**, *S. aureus* 45SNG3 **(H)** and *S. epidermidis* 46SNG3 **(K)** with *L. reuteri* 7SNG3. Representative CLSM images of dual-species biofilms of *K. pneumoniae* 9SNG3 **(C)**, *S. marcescens* 10SNG3 **(F)**, *S. aureus* 45SNG3 **(I)** and *S. epidermidis* 46SNG3 **(L)** with *L. salivarius* 20SNG2. Scale bar: 50 µm.

SEM and CLSM images of the biofilms formed by the four pathogens, either when growing alone or when co-cultured with one of the LAB strains are displayed in **Figures 5** and **6**, respectively. SEM images of *K. pneumoniae* 9SNG3 and *S. marcescens* 10SNG3 biofilms revealed the presence of small aggregates (**Figures 5A, D**) whereas the cells in the staphylococcal biofilms were glued in a mucoid matrix forming grape-like structures, especially in the case of *S. aureus* 45SNG3 (**Figures 5G, J**). Structural parameters of these biofilms were obtained by CLSM imaging. Although both enterobacterial strains shared a structural pattern, the biofilms formed by *K. pneumoniae* 9SNG3 were three times denser and thicker (maximum thickness: 20 µm; biovolume: 4.56 [± 0.53] × 10^5^ µm^3^) than those formed by *S. marcescens *10SNG3 (maximum thickness: 6 µm; biovolume: 1.43 [± 0.07] × 10^5^ µm^3^) (**Table 1**). In contrast, the biofilms formed by the two staphylococcal strains achieved much lower densities (4.37 [± 0.74] × 10^4^ µm^3^ and 2.32 ± [0.21] × 10^4^ µm^3^, for *S. aureus* 45SNG3 and *S. epidermidis* 46SNG3, respectively) and were thinner (maximum width of 7 µm for *S. aureus* 45SNG3 and 3 µm for *S. epidermidis* 46SNG3) (**Table 1**). Structural differences between these two species were also observed by CLSM imaging (**Figure 6**). While *S. epidermidis* 46SNG3 colonized the surface homogenously (**Figure 6J**), *S. aureus *45SNG3 (**Figure 6G**) tended to form dense clusters all over the surface.

When the four nosocomial strains were co-cultured with one of the LAB strains, the structure of the dual-species biofilms was deeply modified with respect to their respective monospecies biofilms. In the presence of *L. salivarius* 20SNG2, the cells of *K. pneumoniae* 9SNG3 and *S. marcescens* 10SNG3 lost their capacity to spread all over the surface (**Figures 6C, F**). Indeed, the biofilm landscape changed from a rather homogeneous cell tapestry to the presence of areas with scattered cells or small cell clusters (**Figure 5**). The maximum width of *K. pneumoniae* 9SNG3*-L. salivarius* 20SNG2 biofilms (20 µm) was almost half reduced compared to the monospecies biofilm of *K. pneumoniae* 10SNG3 (13 µm) (**Table 1**). This phenomenon was also observed when these two pathogens were co-cultured with *L. reuteri *7SNG3. In *L. reuteri *7SNG3*-K. pneumoniae* 9SNG3 biofilms (**Figure 5B**), the ability of *K. pneumoniae* 9SNG3 to colonize the surface in a homogeneous manner was lost. Instead, areas with chains of lactobacilli segregated from scattered *K. pneumoniae* 9SNG3 cells were observed. A similar pattern was observed in the case of *L. reuteri* 7SNG3-*S. marcescens* 10SNG3 biofilms (**Figure 5E**) although in this case *S. marcescens* cells remained closer. In general, co-cultured of these enterobacterial strains with *L. reuteri* 7SNG3 reduced the thickness of the observed structures, with a maximum width average of 12 µm for the *L. reuteri* 7SNG3-*K. pneumoniae* 9SNG3 mix and 6 µm for the *L. reuteri* 7SNG3-*S. marcescens* 10SNG3 one (**Table 1**).

*S. aureus* 45SNG3 and *L. salivarius* 20SNG2 cells were close to each other when these two strains were co-cultured. In some areas, small cocci aggregates were connected to elongated lactobacilli cells through short mucoid threads of matrix. This phenomenon did not happen with *S. epidermidis* 46SNG3 (**Figures 5L**, **6L**), whose ability to colonize the surface was drastically reduced in the presence of *L. salivarius* 20SNG2. Both *L. reuteri* 7SNG3 and *S. aureus* 45SNG3 cells formed aggregates when they were co-cultured (**Figure 5H**). Due to this association, *S. aureus* 45SNG3 cells lost its ability to spread all over the surface, being entrapped into mucoid matrix lumps and partially surrounded by lactobacilli cells. In *L. reuteri *7SNG3-*S. epidermidis *46SNG3 biofilms, both species formed large aggregates surrounded by a large amount of matrix (**Figure 5K**). Some of the cells seemed to be damaged as they showed a distorted morphology (**Figure 5K**). Biovolume values of *L. reuteri *7SNG3-*S. aureus* 45SNG3 and *L. reuteri *7SNG3-*S. epidermidis* 46SNG3 biofilms were approximately two and six times higher, respectively, when compared with their corresponding monospecies biofilms (**Table 1**).

## Discussion

Biofilms can be found colonizing a plethora of ecological niches forming multispecies communities. Interactions among species and strains in these communities can be neutral, antagonistic or synergistic, and they can control the composition and spatial distribution of the species inside the biofilm (Makovcova et al., 2017; Guéneau et al., 2022). In previous works, we showed that the inner surface of NEFTs used for preterm feeding are mostly colonized by some species of the genus *Staphylococcus* and others belonging to the Family *Enterobacteriaceae* while some LAB may also appear, although at a much lower frequency and abundance (Gómez et al., 2016a; Jara et al., 2021). LAB strains well adapted to the inner NEFT environment may be good candidates for coating of these devices in order to minimize the risks associated to the presence and proliferation of relevant nosocomial pathogens.

Both LAB strains, *L. salivarius* 20SNG2 and *L. reuteri* 7SNG3, developed well-structured biofilms as revealed by SEM and CLSM imaging. Although heterogeneous structures in LAB biofilms have been reported (Boles et al., 2004; Neu and Lawrence, 2017; Jara et al., 2020), our strains showed a rather homogenous colonization pattern and were able to cover almost all the surfaces used as adhesion substrates. This colonization was particularly intense at the liquid-air interphase, where desiccation phenomena occur, and it was accompanied by an increase of OD values, suggesting a large production of extracellular matrix that helps cells to stay firmly attached to the surface (Bowen et al., 2018; Karygianni et al., 2020). Such interphases are common in the inner surfaces of NEFTs since the food circulation is discontinuous. In these cases, the feeds (milk or formula) are pumped through the NEFTs and, although most of them reach the stomach, some food residues remain inside the tubes. As preterm are in a supine position, residual food is generally blanketing the bottom part of the NEFT whereas its top part remains empty. This fact can promote adhesion and growing of either the beneficial or pathogenic strains that may contact with these devices.

In order to gain insight into the ecological relationships that prevail when LAB strains share the same niche with nosocomial pathogens, we developed dual-species biofilms combining one of our two LAB strains with one of the four pathogenic strains selected in this study. In terms of cellular density, LAB strains had a neutral or negative impact on the Gram-negative species. Several studies have demonstrated the inhibitory effect of some LAB strains against a wide variety of Gram-negative bacteria, including *Pseudomonas aeruginosa*, *Escherichia coli*, *Klebsiella* spp. and *Enterobacter* spp. (Rybalchenko et al., 2015; Kang et al., 2017; Shokri et al., 2018; Merino et al., 2019; Wu et al., 2022). LAB have developed numerous strategies for competing with other bacteria in natural environments. Among them, the synthesis of antimicrobial compounds with a wide antimicrobial spectrum (organic acids, biosurfactants and bacteriocins, among others) must be highlighted (Salas-Jara et al., 2016; Kang et al., 2017; Englerová et al., 2018; Merino et al., 2019; Guéneau et al., 2022). Competition between LAB and pathogenic species for receptor and nutrients has also been described (Guillier et al., 2008). However, these studies were carried out in liquid media where species interactions are not necessarily similar to those observed in a biofilm model. For instance, Kang et al. (2017) demonstrated that *Limosilactobacillus fermentum* and *L. salivarius* strains had antimicrobial activity towards methicillin-resistant *S. aureus* (MRSA) strains in a planktonic co-culture assay whereas only *L. salivarius* retained antimicrobial activity in a dual species biofilm simulation. Similar studies have shown that the use of certain strains of *Lacticaseibacillus casei, L. reuteri*, *Lactiplantibacillus plantarum* or *Lentilactobacillus kefiri* can reduce the colonization ability of *Streptococcus mutants*, *Staphylococcus* spp., *E. coli* and *Salmonella* spp. to the surface of teeth, food-contact surfaces and medical devices (Rybalchenko et al., 2015; Wu et al., 2015; Kang et al., 2017; Shokri et al., 2018; Wasfi et al., 2018; Merino et al., 2019; Wu et al., 2022).

Loss in attached cell density due to interspecies interaction when the enterobacterial strains were forming dual biofilms with the LAB strains was confirmed by a significant reduction in biomass (cells + EPS) compared with their respective monospecies biofilms. Such reduction may be the result of a lower cell density and/or a lower synthesis of EPS, which is considered as a virulence factor when produced by pathogenic bacteria (Lepargneur and Rousseau, 2002; Salas-Jara et al., 2016; Jara et al., 2020). It has been already reported that *S. mutans* produced less EPS when forming biofilms with two strains of *L. salivarius* (Wu et al., 2015). In fact, the expression of some virulence factors can be modulated due to interspecies interactions (Høiby et al., 2011; Karygianni et al., 2020). In this sense, it has been described that biofilm formation by *S. mutants* ATCC 25175 and *Gardenella vaginalis* ATCC49145 are impaired in the presence of LAB strains because of the downregulation of the genes *gtfs* and *ftf* (glucosyltransferase and fructosyltransferase-related genes) (Wasfi et al., 2018; Qian et al., 2021). Similarly, the genes *gtfB*, *gtfC* and *ftf* of *S. mutans* 22 and *S. mutans* ATCC 35668 were downregulated by biosurfactants produced by *Lacticaseibacillus rhamnosus* (Tahmourespour et al., 2019). Moreover, *L. casei* and *L. acidophilus* can modulate the expression of *luxS*, a gene related to biofilm formation and matrix production in a large number of microorganisms (Lemos et al., 2004; Ahmed et al., 2014). Therefore, our LAB strains and/or their metabolites could have interfered with the ability of *K. pneumoniae* 9SNG3 and *S. marcescens* 10SNG3 to form dense biofilms by reducing the amount of matrix that they produce when they are alone.

These results were confirmed by SEM imaging. The dual species biofilms of *K. pneumoniae* 9SNG3 and *S. marcescens* 10SNG3 with any of the LAB strains showed that both species were sharing the same niche but did not tend to aggregate. In addition, the biofilm thickness and biovolume of the biofilms formed by the enterobacterial strains in pure monocultures decreased when they interacted with the LAB strains. Competition between microorganisms that are sharing the same surface can lead to structural changes that favored one species over others (Graver and Wade, 2011; Makovcova et al., 2017). SEM images showed that the cells of some of the pathogenic strains displayed an abnormal morphology, suggesting the ability of the LAB strains to damage and/or kill potential competitors. This finding has already been described for the interactions between *L. salivarius* CGMCC207 and *S. aureus* (Li et al., 2021).

It must be highlighted that we observed a positive relationship between *L. reuteri* 7SNG3 and *S. aureus* 45SNG3 (accompanied by a stimulation of matrix production by the staphylococcal strain) and, also, between *L. salivarius* 20SNG3 and *S. epidermidis* 46SNG3. This phenomenon has been previously described and it must be taken into account when selecting strains for practical applications. Jara et al. (2020) demonstrated that a surface conditioned by a preformed probiotic biofilm improved *Listeria monocytogenes* Scott A adhesion. Moreover, SEM images showed these species were totally interconnected to each other (forming aggregates) and trapped into an amorphous matrix. This type of amorphous matrix has been previously described in *S. aureus* biofilms that are enriched in exopolysaccharides (Wang et al., 2015; Makovcova et al., 2017).

Currently, the use of some LAB strains to minimize the colonization of surfaces by potentially pathogenic species is gaining attention (Stone et al., 2020; D’Accolti et al., 2022). The colonization of clinical devices, as NEFTs, by strains of *K. pneumoniae* and *S. marcescens* is especially relevant as these microorganisms are frequently associated with outbreaks in NICUs (Marchant et al., 2013; Escribano et al., 2019; Moles et al., 2019; Chen et al., 2020; Hassuna et al., 2020; Joubert et al., 2022). The knowledge provided by this study would guide the selection of potentially probiotic strains that are already sharing the same niche with the pathogens for future applications in Neonatology. These strains would be supplied together with the food enabling the establishment of a healthier microbial community inside NEFTs.

## Data availability statement

The raw data supporting the conclusions of this article will be made available by the authors, without undue reservation.

## Author contributions

BO and JMR designed research; JJ, RJ, VA-V, LF, and IL-M performed research; JJ, JMR, and BO wrote the paper. All authors contributed to the article and approved the submitted version.

## Funding

This work was supported by the project PID2019-105606RB-I00 financed by the Spanish Ministry of Economy and Competitiveness.

## Acknowledgments

JJ is recipient of a fellowship financed by the Spanish Ministry of Education (Ref. FPU18/02433) and RJ acknowledges financial support through grant PRE2020-096035 of the Spanish Ministry of Science and Innovation.

## Conflict of interest

The authors declare that the research was conducted in the absence of any commercial or financial relationships that could be construed as a potential conflict of interest.

The reviewer RC declared a past co-authorship with the author JR to the handling editor.

## Publisher’s note

All claims expressed in this article are solely those of the authors and do not necessarily represent those of their affiliated organizations, or those of the publisher, the editors and the reviewers. Any product that may be evaluated in this article, or claim that may be made by its manufacturer, is not guaranteed or endorsed by the publisher.

## Supplementary material

Supplementary Figure 1 Experimental system for biofilm development. Reprinted from Food Control, 65, 143-151. Puga, C. H., SanJose, C., & Orgaz, B. (2016). Biofilm development at low temperatures enhances Listeria monocytogenes resistance to chitosan. Copyright (2016), with permission from Elsevier.
