# Supplementary figures and images for: Interspecies relationships between nosocomial pathogens associated to preterm infants and lactic acid bacteria in dual-species biofilms
Source: Front Cell Infect Microbiol. 2022 Oct 17;12:1038253. doi: 10.3389/fcimb.2022.1038253 (PMC9618709; doi:10.3389/fcimb.2022.1038253)

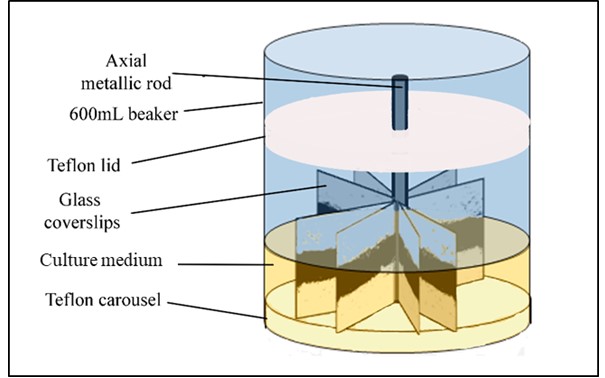

Supplement: Supplementary Figure 1 — Experimental system for biofilm development. Reprinted from Food Control, 65, 143-151. Puga, C. H., SanJose, C., & Orgaz, B. (2016). Biofilm development at low temperatures enhances Listeria monocytogenes resistance to chitosan. Copyright (2016), with permission from Elsevier. [file Image_1.jpeg]
